# Anatomical considerations for appropriate mini-plate positioning in open-door laminoplasty to avoid plate impingement and screw facet violation

**DOI:** 10.1038/s41598-022-09434-z

**Published:** 2022-04-01

**Authors:** Jae Jun Yang, Sehan Park

**Affiliations:** Department of Orthopedic Surgery, Dongguk University Ilsan Hospital, 14 Siksadong, Ilsandong-gu, Goyang-si, Gyeonggi-do 411-773 Republic of Korea

**Keywords:** Risk factors, Musculoskeletal system

## Abstract

This study aimed to describe a safe zone for mini-plate positioning that can avoid instrument-related complications in laminoplasty. Fifty-one patients who underwent laminoplasty and were followed up for at least 1 year were retrospectively reviewed. The posterior surface length and inferior pole angle of the lateral mass were measured at each level using computed tomography. The safe zone was defined based on these measurements. Incidences of screw facet violation and plate impingement were recorded. Patient-reported outcome measures were compared between the appropriate position (AP) and inappropriate position (IP) groups. Among 40 patients included, 15 (37.5%) had inappropriate plate positioning, causing screw facet violation or plate impingement, which more commonly occurred at distal (C5, C6) and proximal (C3, C4) levels, respectively. Lateral mass posterior surface length was shorter at the proximal levels, and the inferior pole angle of the lateral mass was smaller at the distal levels, signifying that the lateral mass became thin and long at the distal levels. Patient-reported outcome measures were not significantly different between the two groups. However, cervical range of motion at the final follow-up was significantly less in the IP group (*p* = 0.01). The suggested safe zone demonstrates that inserting the mini-plate with plate-to-lateral mass inferior pole distances of 4–5 mm and 5–6 mm at the C3–C5 and C6–C7 levels, respectively, would avoid instrument-related complications. The risk of plate impingement was higher at the proximal level, whereas the risk of screw facet violation was higher at the distal level in open-door cervical laminoplasty. These risks coincide with anatomical differences at each level. Despite inappropriate positioning of the mini-plate, clinical outcomes were not adversely affected.

## Introduction

Open-door laminoplasty is a safe and widely applied surgical technique for the treatment of cervical myelopathy^1,2^. Various instruments have been used to maintain the opening of the lamina, including bone block, stay suture, wire, and mini-plate^3^. Mini-plate fixation has been reported to reduce the risk of lamina reclosure while enhancing bone healing at the trough side^4,5^. Fixation of the mini-plate is generally a safe procedure, and critical complications related to its instrumentation are rare since it is fixed with short screws^5,6^.

Although mini-plate fixation with screws is free from adverse complications such as vertebral artery injury or nerve root irritation, which could occur with lateral mass screw or pedicle screw fixation, instrument-related complications can still occur^3,7–9^. Considering the anatomy of the lateral mass, mini-plates located too caudally would result in screw facet violation. However, when the plate is located excessively cranially, the inserted plate would impinge on the lateral mass at the proximal level. Previous studies have demonstrated that screw facet violation is not rare in mini-plate fixation for laminoplasty, where incidence ranges from 34.1 to 37.4%^3,8,9^. Screw facet joint violation or plate impingement caused by the mini-plate raises concern for possible facet degeneration and postoperative neck pain^9^. Aggravation of neck pain and kyphosis after laminoplasty are common, possibly caused by instrument positioning^10–12^. It has also been reported that facet joint violation by mini-screws can decrease cervical range of motion (ROM)^9^.

While previous studies have described optimal insertion points and trajectories for lateral mass screws or pedicle screws, not many have demonstrated an optimal method for mini-plate fixation for laminoplasty^3,8,13,14^. Therefore, this study was conducted to (1) describe the incidence of screw facet joint violation and plate impingement in open-door laminoplasty using mini-plate fixation, (2) define a safe zone to avoid instrument-related complications, and (3) identify whether inappropriately positioned mini-plates would adversely affect clinical outcomes.

## Materials and methods

### Study design and participants

This was a retrospective cohort study approved by the institutional review board of our institute (Dongguk University Ilsan Hospital Institutional Review Board 2021-09-020). All methods were carried out in accordance with relevant guidelines and regulations. Informed consent was waived owing to the study’s retrospective nature. The study was conducted in accordance with the Strengthening the Reporting of Observational Studies in Epidemiology (STROBE) statement for cohort studies.

The medical records of 51 patients who underwent laminoplasty for cervical myelopathy caused by spondylosis or ossification of the posterior longitudinal ligament between September 2012 and March 2019 were retrospectively reviewed. Patients (1) who underwent surgery due to trauma, infection, or tumor; (2) who lacked radiographic or clinical data; and (3) who had a follow-up period of less than 1 year were excluded.

Patients with screw facet joint violation or possible plate impingement with cranial lateral mass observed on postoperative computed tomography (CT) were classified into the inappropriate position patient group (IP group). Patients with no identifiable mini-plate-related complications were defined as the appropriate position patient group (AP group).

### Surgical technique

Patients were placed in a prone position with their heads located on the Mayfield headrest. A midline posterior approach was used to expose the spinous process and lamina of the indicated levels. Dissection was performed until the lamina-lateral mass junction was exposed. The spinous processes were resected at the base. Open-side and hinge-side troughs were made at the lamina-lateral mass junction. After the lamina was carefully opened to avoid complete fracture of the hinge side, a mini-plate (Centerpiece, Medtronic, Minneapolis, MN, USA) was used to maintain the lamina opening. We attempted to position the mini-plate at the center of the posterior surface of the lateral mass. Two 5-mm screws were used to fix the plate at the lamina, and two 5-mm screws were inserted at the lateral mass to anchor the plate. Screws into the lateral mass were inserted perpendicular to the posterior surface of the lateral mass. For C7, partial laminectomy rather than open-door laminoplasty was performed to preserve muscle insertion in the spinous process^15^.

### Variables and radiographic measurements

The neck pain visual analog scale (VAS), arm pain VAS, and neck disability index (NDI) were recorded preoperatively and at each postoperative follow-up. Three-dimensional CT scans were taken preoperatively for surgical planning and at 2 days postoperatively to evaluate adequate decompression and instrument position^16^.

**Figure 1:**
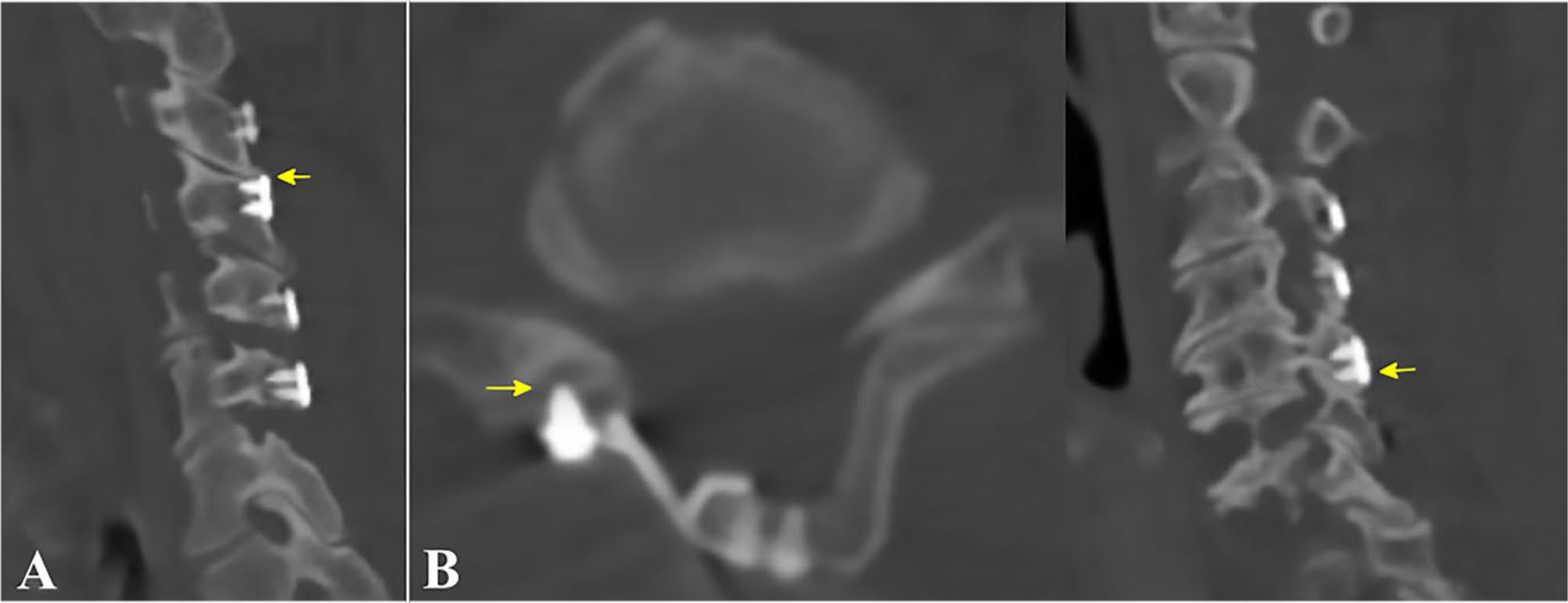
Inappropriate plate positioning. (**A**) Cranially located mini-plate causes impingement with the lateral mass of the proximal adjacent level (arrow). (**B**) Screw facet joint violation is detected in axial and sagittal computed tomography images when the mini-plate is located too caudally (arrows).

Possible plate impingement was diagnosed when the cranial edge of the mini-plate reached the caudal edge of the lateral mass of adjacent proximal level (Fig. 1A). Screw facet violation was defined as the screw penetrating the ventral surface of the lateral mass detected on axial or sagittal reconstructed CT images (Fig. 1B).

**Figure 2:**
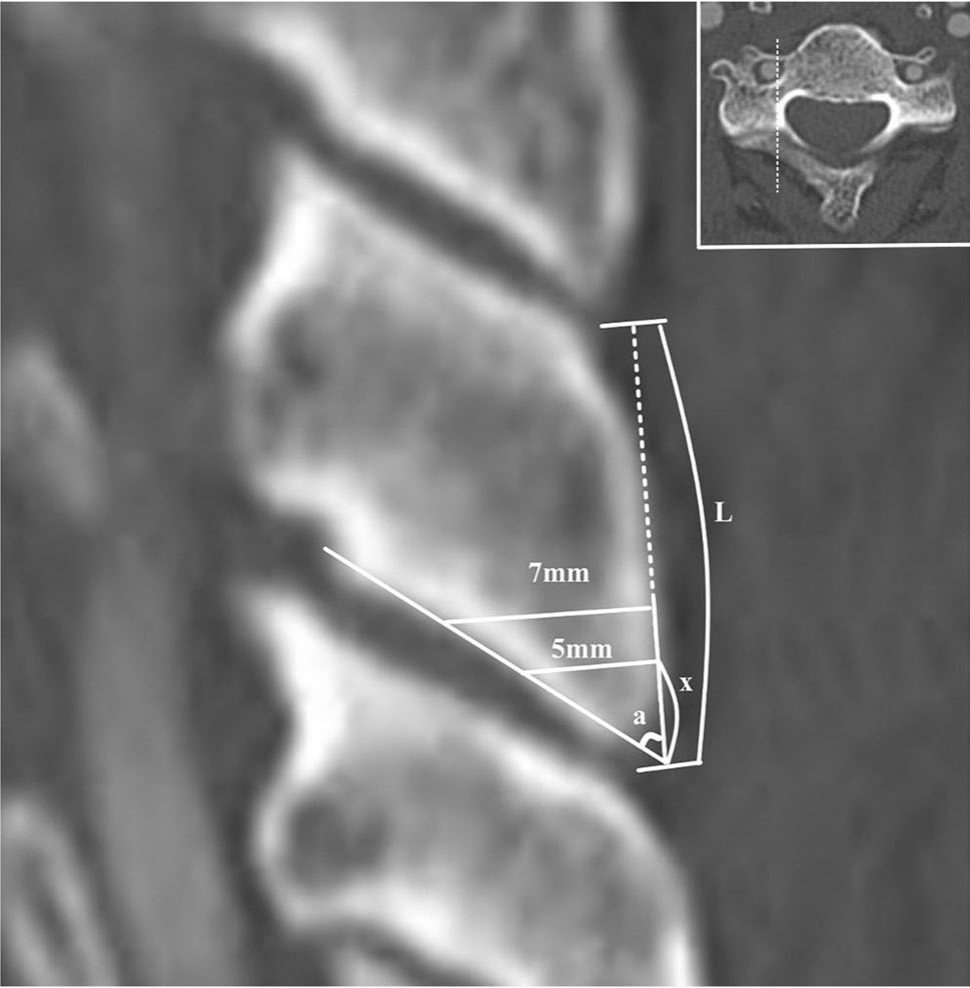
Radiological measurement. The posterior surface length of the lateral mass (L) is measured as the distance between the cranial edge and caudal edge of the lateral mass in sagittal computed tomography images. The inferior pole angle of the lateral mass (a) is measured as the angle between the line drawn through the posterior surface of the lateral mass and the line drawn through the ventral-inferior border of the lateral mass that compose the facet joint. The minimum distance for screw placement (x) is calculated as the inferior pole angle (a) and the length of the screw (5 or 7 mm) using the following equation: [x = length of screw ÷ tangent (a)].

The posterior surface length of the lateral mass was measured as the distance between the cranial and caudal edges of the lateral mass on sagittal CT images. A sagittal image showing a pedicle-lateral mass junction was selected because the mini-plate is usually fixed to the lateral mass at this location. Within the same sagittal image, the inferior pole angle of the lateral mass was measured as the angle between the line drawn through the posterior surface of the lateral mass and the line drawn through the ventral-inferior border of the lateral mass that composed the facet joint. Measurements were performed bilaterally, and the mean value was used for evaluation (Fig. 2).

Cervical lordosis was measured in the lateral view in the neutral position based on the angle between the lines passing through the lower margin of C2 and C6 or C7. The sagittal vertical axis (SVA) of C2-C7 was defined as the horizontal distance between the vertical line from the center of C2 and the posterior-superior aspect of C7. Cervical ROM was measured as the change in the angle between the lower margin of C2 and the lower margin of C7 on dynamic (flexion and extension) radiographs.

### Definition of safe zone

**Figure 3:**
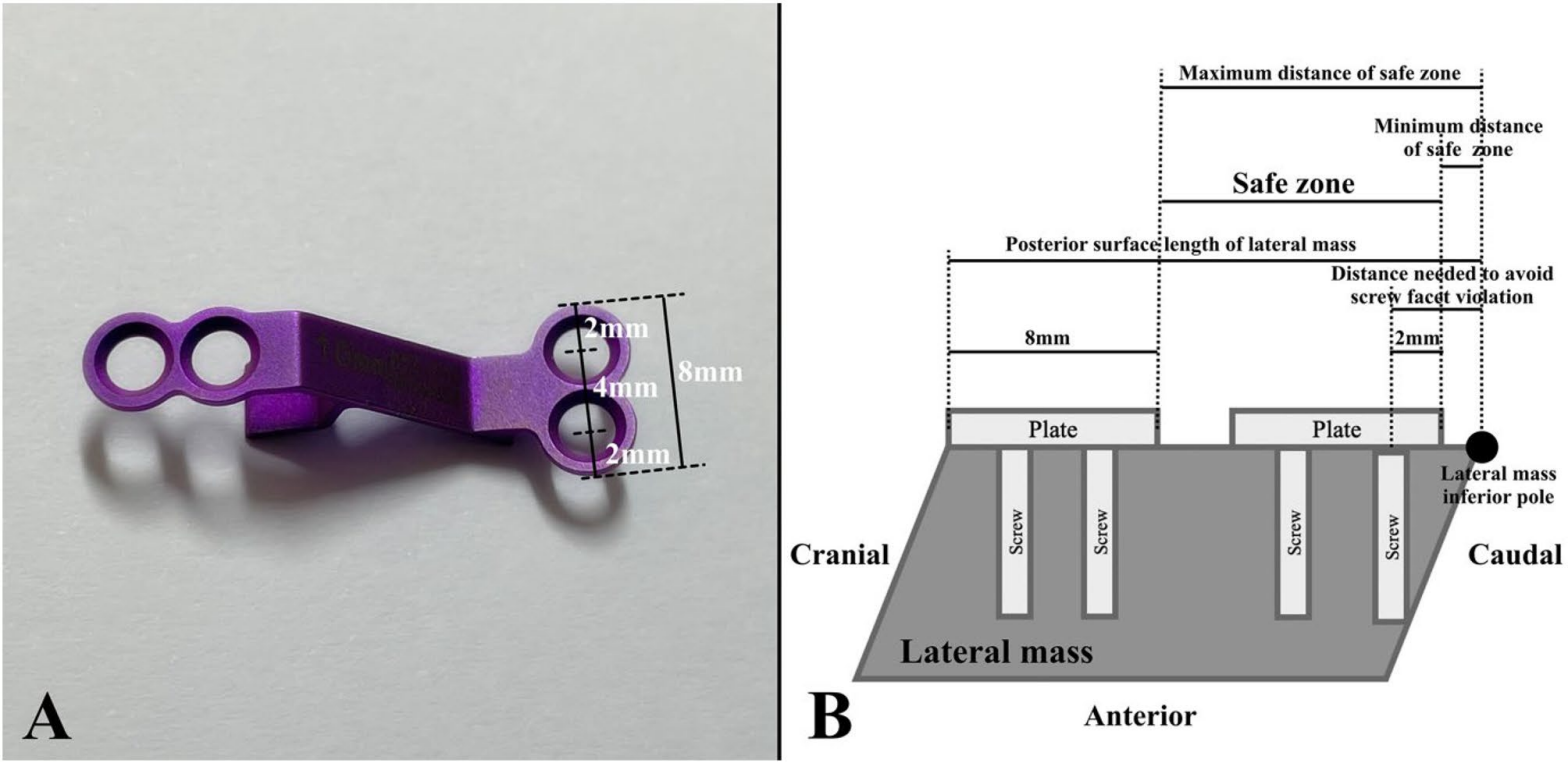
Defining the safe zone. (**A**) Measurement of the laminoplasty mini-plate. The distances between the edge of the plate and the center of the screw fixation hole, between the center of each screw fixation hole, and between the cranial and caudal edges of the plate are 2 mm, 4 mm, and 8 mm, respectively. (**B**) Defining the safe zone based on the measurement. The minimum distance of the safe zone would be [distance needed to avoid screw facet violation (x) – 2] where the plate caudal edge-to-plate caudal screw hole center distance is 2 mm. The maximum distance of the safe zone would be [lateral mass posterior surface length – 8] where the plate cranial edge-to-plate caudal edge distance is 8 mm.

A safe zone that can avoid both screw facet violation and plate impingement at the proximal adjacent level was defined. To define the safe zone, we first measured the size of the mini-plate (Centerpiece, Medtronic, Minneapolis, MN, USA) used for all patients included in the study. The distances between the edge of the plate and the center of the screw fixation hole, between the center of each screw fixation hole, and between the cranial and caudal edges of the plate were 2 mm, 4 mm, and 8 mm, respectively (Fig. 3A).

Second, the minimum distance from the inferior pole of the lateral mass needed to avoid screw facet violation was calculated. The minimum distance for screw placement (x) was calculated using the inferior pole angle (a) and the length of the screw using the following equation: [x = length of screw ÷ tangent (a)] where tangent (a) would be the same as [length of screw ÷ x] (Fig. 2).

Finally, a safe zone that could avoid both screw facet violation and plate impingement was defined as the distance between the caudal edge of the mini-plate and the inferior pole of the lateral mass. We assumed that the screw was inserted perpendicular to the posterior surface of the lateral mass. The minimum distance of the safe zone would be [distance needed to avoid screw facet violation (x) – 2] where the plate caudal edge-to-plate caudal screw hole center distance is 2 mm. The maximum distance of the safe zone would be [lateral mass posterior surface length – 8] since the plate cranial edge-to-plate caudal edge distance is 8 mm (Fig. 3B).

### Statistical analysis

Student’s t-test was performed to compare the patient-reported outcome measure results between the IP and AP groups. Paired t-test was performed to compare preoperative and postoperative values. The Mann–Whitney U test and Wilcoxon signed rank test were used to analyze cervical sagittal alignment and ROM, as these parameters did not demonstrate a normal distribution according to the Shapiro–Wilk test. All data management and statistical analyses were performed using SPSS version 21.0 software (SPSS Inc., Chicago, IL, USA). Statistical significance was set at *p* < 0.05.

## Results

### Patient characteristics and inappropriate plate positioning

Forty patients with 120 levels met the inclusion criteria and were included in the study. Screw facet violation was observed in 8 patients (20.0%) and in 8 levels (6.7%). Furthermore, plate impingement was detected in 9 patients (22.5%) and in 11 levels (9.2%). In total, 15 patients (37.5%) had inappropriate plate positioning and were classified as the IP group (age, 67.7 ± 9.8 years; male, 76.2%; follow-up, 75.0% [15/20]). The remaining 25 patients (62.5%) were classified into the AP group (age, 62.1 ± 11.6 years; male, 76.2%; follow-up, 80.6% [25/31]). One patient (4.0%) in the AP group demonstrated adjacent segment degeneration during follow-up, which required anterior operation revision. Baseline patient characteristics were not significantly different between the AP and IP groups (Table 1).

**Table 1:** Patient characteristics.

	AP group	IP group	*P* value
N	25	15	
**Sex**			
Male	16 (76.2%)	8 (72.7%)	1.00
Female	5 (23.8%)	3 (27.3%)	
Age	62.1 ± 11.6	67.7 ± 9.8	0.25
Follow-up (m)	19.0 ± 20.9	20.2 ± 16.7	0.63
Number of levels operated	4.1 ± 1.1	4.7 ± 1.1	0.22

*AP* appropriately positioned, *IP* inappropriately positioned, *m* months.

Sex was analyzed using a chi-square test.

Age, follow-up period, number of levels operated were analyzed using student’s t-test.

**Figure 4:**
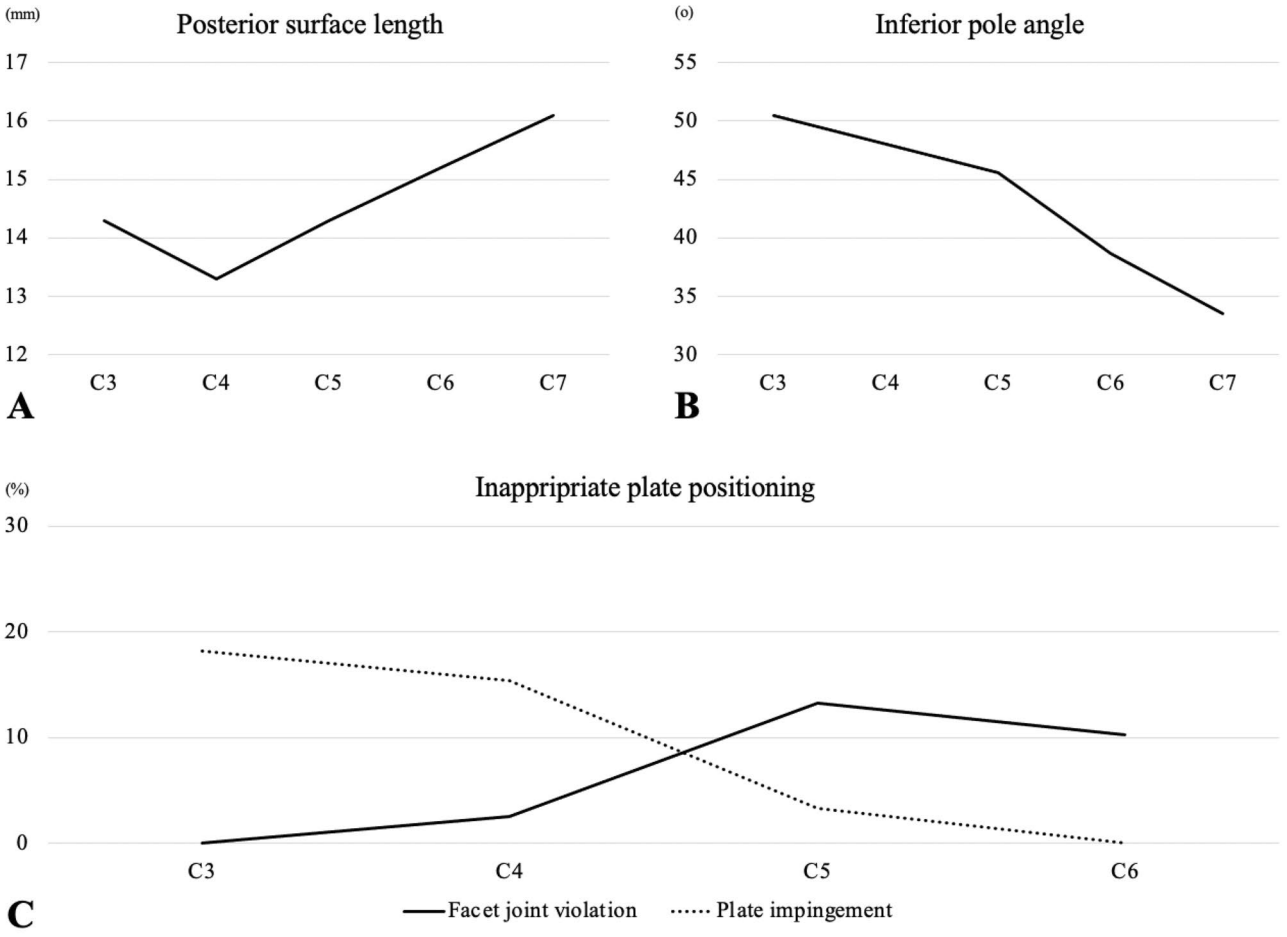
Radiographic results. (**A**) Lateral mass posterior surface length at each level. (**B**) Lateral mass inferior pole angle at each level. (**C**) Incidence of screw facet joint violation and plate impingement at each level.

Screw facet joint violation was more frequently observed at the distal levels, including C5 and C6, whereas no screw violation was observed in C3. In contrast, plate impingement was more frequently detected at the proximal levels including C3 and C4, whereas no plate impingement was detected at the most caudal level, C6 (Table 2) (Fig. 4). No plate/screw pullout or breakage occurred.

**Table 2:** Inappropriate plate positioning.

	Facet joint violation	Plate impingement
C3	0% (0/22)	18.2% (4/22)
C4	2.6% (1/39)	15.4% (6/39)
C5	13.3% (4/30)	3.3% (1/30)
C6	10.3% (3/29)	0% (0/29)

### Radiographic results

The lateral mass posterior surface length was longer at the distal levels. Furthermore, the inferior pole angle of the lateral mass tended to decrease at these levels. Because of the smaller inferior pole angle at the distal levels, the calculated minimum distance from the inferior pole to insert screws by [x = length of screw ÷ tangent (a)] was higher at the distal levels (Table 3) (Fig. 4).

**Table 3:** Radiographic measurements.

Levels	Posterior surface length (mm)	Inferior pole angle (°)	Distance from inferior pole needed to avoid screw facet violation (5 mm)	Distance from inferior pole needed to avoid screw facet violation (7 mm)
C3	14.3 ± 1.9	50.5 ± 4.8	4.2 ± 0.7	5.9 ± 1.0
C4	13.3 ± 2.1	48.0 ± 5.4	4.6 ± 0.9	6.5 ± 1.2
C5	14.3 ± 1.5	45.6 ± 4.9	5.0 ± 0.9	7.0 ± 1.2
C6	15.2 ± 1.6	38.7 ± 4.9	6.4 ± 1.2	9.0 ± 1.6
C7	16.1 ± 1.6	33.5 ± 4.3	7.7 ± 1.3	10.8 ± 1.8

Distance from the in inferior pole needed to avoid screw facet violation was calculated by [length of screw ÷ tangent (inferior pole angle)].

Lordosis of C2-C7 did not demonstrate significant intergroup differences during any postoperative follow-up periods. Furthermore, there was no significant difference in C2-C7 SVA between the AP and IP groups. However, cervical ROM significantly decreased at the final follow-up in the IP group (*p* < 0.01), while it did not change significantly in the AP group (*p* = 0.91). Cervical ROM at the final follow-up was significantly smaller in the IP group than in the AP group (*p* < 0.01) (Table 4).

**Table 4:** Cervical sagittal alignment and range of motion.

		AP group	IP group	*P* value
C2-C7 lordosis (°)	Preoperative	16.8 ± 12.0	8.8 ± 7.1	0.04*
	Post op 6w	14.1 ± 14.9	9.0 ± 8.0	0.31
	Final follow-up	13.2 ± 11.4	5.7 ± 10.6	0.06
C2-C7 SVA (°)	Preoperative	19.1 ± 8.4	19.9 ± 16.2	0.64
	Post op 6w	19.6 ± 19.8	22.4 ± 14.8	0.41
	Final follow-up	21.4 ± 11.9	26.3 ± 15.9	0.51
ROM (°)	Preoperative	33.8 ± 11.0	34.1 ± 11.0	0.85
	Post op 6w	33.2 ± 10.9	24.2 ± 10.6	0.05
	Final follow-up	33.5 ± 11.2	20.4 ± 12.1	0.01*

*AP* appropriately positioned, *IP* inappropriately positioned, *SVA* sagittal vertical axis, *ROM* range of motion, *w* weeks.

Comparison between two groups were performed using Mann–Whitney U test.

*P < 0.05.

Among the 40 patients included, 25 (62.5%) underwent a 1-year follow-up CT, which enabled evaluation of hinge site union status. Among the 76 segments evaluated, three (3.9%) demonstrated hinge site non-union. One segment was associated with screw facet joint violation, while the other two segments were not associated with screw violation or plate impingement.

### Patient-reported outcome measures

Neck pain VAS, arm pain VAS, and NDI significantly improved after the operation in both groups (*P* < 0.01). No significant difference in patient-reported outcome measures between the AP and IP groups was observed at each follow-up period (Table 5).

**Table 5:** Patient reported outcome measures.

		AP group	IP group	*P* value
Neck pain VAS	Preoperative	6.1 ± 2.6	6.2 ± 2.1	0.91
	Post op 6w	2.2 ± 2.1	1.7 ± 2.0	0.46
	Post op 1y	1.2 ± 1.3	0.6 ± 1.3	0.26
	Final follow-up	1.6 ± 1.3	2.0 ± 3.1	0.46
Arm pain VAS	Preoperative	7.6 ± 1.1	8.4 ± 0.8	0.06
	Post op 6w	2.6 ± 2.2	3.6 ± 2.1	0.13
	Post op 1y	1.5 ± 1.6	1.6 ± 1.8	0.94
	Final follow-up	1.6 ± 1.1	2.0 ± 3.1	0.88
NDI	Preoperative	25.3 ± 5.9	27.7 ± 7.3	0.57
	Post op 6w	13.4 ± 7.0	14.8 ± 9.6	0.92
	Post op 1y	11.2 ± 9.9	7.8 ± 5.0	0.77
	Final follow-up	11.9 ± 10.1	14.8 ± 7.1	0.95

*AP* appropriately positioned, *IP* inappropriately positioned, *VAS* visual analogue scale, *NDI* neck disability index, *w* weeks, *y* years.

Comparisons between two groups were performed using student’s t-test.

### Suggested safe zone

**Figure 5:**
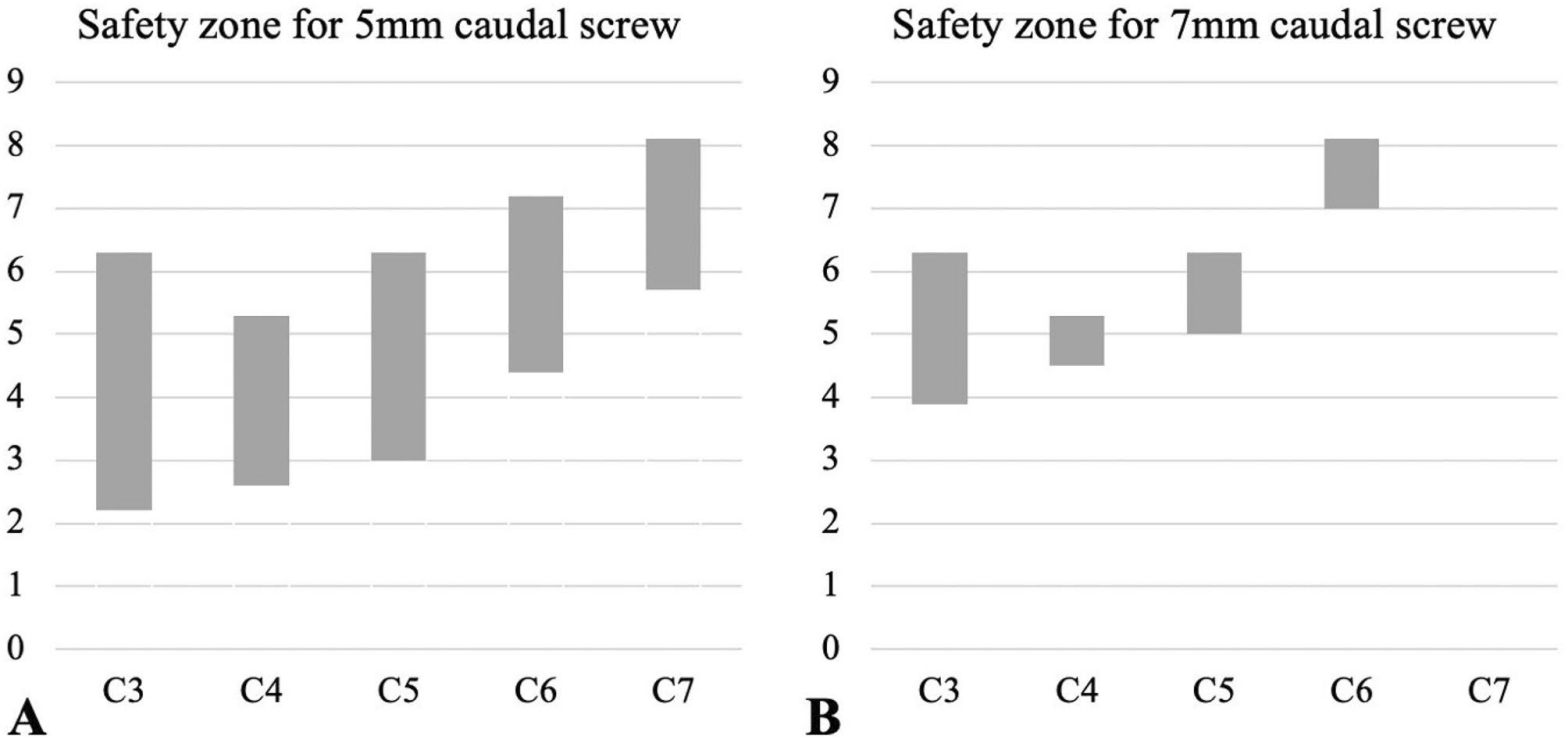
Suggested safe zone. The graph demonstrates the minimum and maximum distances between the caudal edge of the mini-plate and the inferior pole of the lateral mass where the plate can be safely placed.

The suggested safe zone for appropriate plate positioning in laminoplasty is summarized in Table 6 and Fig. 5. When inserting a 5-mm screw for the caudal screw for mini-plate lateral mass fixation, 2- to 3-mm distancing of the plate from the inferior pole of the lateral mass was required for the C3, C4, and C5 levels. However, at the C6 or C7 levels, a distance of approximately 5 mm from the inferior pole of the lateral mass was needed to avoid screw facet violation. Distancing the mini-plate by more than 5–6 mm at C3–C5 and 7–8 mm at C6–C7 from the inferior pole of the lateral mass was not safe because it would cause plate impingement at the cranial level. The safe zone was narrower when inserting a 7-mm screw for the caudal screw as more distance is needed to avoid screw facet violation. Inserting a 7-mm screw for the caudal screw at the C7 level leaves no safe zone due to the thin lateral mass at this level, as demonstrated by the small inferior pole angle at C7.

**Table 6:** Suggested safe zone defined by the distance between inferior pole of lateral mass and caudal edge of mini-plate.

Levels	Distance between inferior pole of lateral mass and caudal edge of plate (mm)					
	5 mm screw for caudal screw			7 mm screw for caudal screw		
	Minimum	Maximum	Median	Minimum	Maximum	Median
C3	2.2	6.3	4.3	3.9	6.3	5.1
C4	2.6	5.3	4.0	4.5	5.3	4.9
C5	3.0	6.3	4.7	5.0	6.3	5.7
C6	4.4	7.2	5.8	7.0	7.2	7.1
C7	5.7	8.1	6.9			

Minimum distance was calculated by [Distance from in inferior pole needed to avoid screw facet violation - 2].

Maximum distance was calculated by [Posterior surface length -8].

Considering the median value of the safe zone, for C3–C5, leaving a 4-to 5-mm distance when inserting a 5-mm screw and distancing the mini-plate 5–6 mm when inserting a 7-mm screw from the inferior pole would avoid both screw facet violation and plate impingement. For C6–C7, leaving 6–7 mm when inserting a 5-mm screw would avoid instrument-related complications. However, for C6–C7, 7-mm screw insertion for caudal screw in mini-plate fixation would be considered unsafe because of the narrow or no safe zone.

## Discussion

Optimal mini-plate insertion for cervical laminoplasty would adequately prevent the reclosure of an open hinge without instrument-related complications such as screw pullout, plate breakage, screw facet violation, and plate impingement with approximate level^3,5,8,17^. Several studies have demonstrated that aggravation of kyphosis, decreased ROM, and postoperative neck pain are common after laminoplasty^10–12,18^. Although injuries to the posterior neck musculature have been commonly discussed as a factor causing these adverse outcomes, inappropriate instrument positioning such as plate impingement or screw facet violation would also have a negative effect on axial symptoms and may have been underestimated^3,8,9^. While transfacet fixation has been reported as a viable technique for fusion, screw facet violation by mini-screws does not limit facet joint motion and would accelerate the degenerative process at the involved level^19^. Chen et al. demonstrated that screw facet joint violation during mini-plate fixation results in decreased ROM and aggravated neck pain, although neurological recovery was not affected^9^.

Two previous studies have suggested safe mini-screw insertion points for laminoplasty^3,8^. Chen et al. demonstrated the safe zone using 3D image rendering^8^. However, the safe zone definition in this study is complex considering that intraoperatively, surgeons can only adjust the plate position in the cranial or caudal direction. Min et al. also demonstrated the minimal safe distance of mini-screw insertion^3^. The limitation of this study is that the measuring method has not been objectively described. Furthermore, although these two studies suggest that a certain distance is needed from the inferior pole of the lateral mass to avoid screw facet violation, they did not consider the possibility of plate impingement when the plate is located too cranially^3,8^. Therefore, the present study attempted to define the safe zone of mini-plate placement by considering both minimum (to avoid screw facet violation) and maximum distances (to avoid plate impingement) from the inferior pole of the lateral mass.

In this study, screw facet violation was more common at the distal levels, including C5 and C6, whereas plate impingement was more common at the proximal levels, such as C3 and C4. The results of radiological measurements demonstrate that this trend is consistent with the anatomical differences between each level. The possibility of plate impingement would be higher at the proximal level because the posterior surface length is shorter at these levels. However, a smaller inferior pole angle at the distal level signifies a thin lateral mass at these levels, which increases the possibility of screw facet joint violation. Considering such anatomical differences at each level, locating the mini-plate more caudally at the proximal levels and more cranially at the distal levels would help avoid instrument-related complications.

The safe zone was described based on the distance between the inferior pole of the lateral mass and the caudal edge of the mini-plate. Although previous reports have used the screw insertion area as the reference point, we used the caudal edge of the mini-plate because it is easier to identify intraoperatively. The suggested safe zone demonstrates that inserting the mini-plate with a plate-to-lateral mass inferior pole distance of 4–5 mm for the C3–C5 levels and 5–6 mm for the C6–C7 levels would avoid instrument-related complications. Min et al. also demonstrated that more distance from the inferior border of the lateral mass is needed at distal levels to avoid screw facet joint violation^3^. It is known that the lateral mass is generally thin at C7, which makes pedicle screw a more preferred choice than lateral mass screw^20,21^. The results of the present study also demonstrated that inserting a 7-mm screw for mini-plate fixation in C6–C7 would be unsafe owing to the thin lateral mass at these levels as demonstrated by the small inferior pole angle. This finding supports performing partial laminectomy rather than laminoplasty at C7 due to a higher chance of instrument-related complications at this level^21,22^.

Laminoplasty is often performed in patients with cervical spondylosis, which distorts the anatomical landmarks due to bony spurs and spondylolisthesis. Lee et al. demonstrated that screw facet joint violation is more common in severely degenerative cervical spine than in mildly degenerative spine^7^. Although the suggested safe zone in the present study could be used as a reference while placing the mini-plate, such distortion of anatomic landmarks would make it difficult to identify appropriate insertion points. Therefore, individual assessment and preoperative planning with radiographic measurements used in this study would further enhance the safety of laminoplasty.

Although shorter screws can prevent facet joint violation, weak fixation strength caused by decreased screw length can lead to screw pull-out. While this phenomenon was not observed in the present study, previous reports have demonstrated that screw pull-out can occur even with 5-mm screw fixation^5^. Park et al. recommended using two screws with relatively longer screw length for lateral masses in order to increase resistance to output force^17^. Therefore, fixation with a longer screw at the optimal area is needed, rather than a decrease in the screw length.

The results of the present study demonstrate that clinical results, such as neck pasin VAS or NDI, were not adversely by affected screw facet joint violation or plate impingement. However, cervical ROM was adversely affected by inappropriate positioning of the instrument. This corresponds to the findings of Chen et al., which suggested that screw facet joint violation is related to decreased ROM^9^. However, both studies included a small number of patients with instrument-related complications, which warrants further evaluation.

This study had several limitations. First, the lateral mass posterior surface is not a plane surface, but rather has a round curvature, and the measuring method of the present study would have limitations reflecting such curved surfaces. However, within the confinement of using two-dimensional images, the round curvature of the lateral mass cannot be completely measured. Furthermore, the minimal safety distance to avoid screw facet violation demonstrated in this study corresponds to that reported in other previous reports^3,8^. Second, as previously discussed, the study has limited capacity to demonstrate the clinical impact of inappropriate instrument positioning due to the small sample size. Finally, the study is not free from the possibility of selection bias because it was a retrospective, single-center study.

In conclusion, the risk of plate impingement was higher at the proximal level, whereas the risk of screw facet violation was higher at the distal level in open-door cervical laminoplasty. These risks coincide with the anatomical differences at each level. The demonstrated safe zone can be used as a reference for plate positioning. Despite inappropriate positioning of the mini-plate, the clinical outcomes were not adversely affected.

## Data Availability

The datasets generated during and/or analyzed during the current study are available from the corresponding author on reasonable request.
